# Identification and Validation of Novel Contraction-Regulated Myokines Released from Primary Human Skeletal Muscle Cells

**DOI:** 10.1371/journal.pone.0062008

**Published:** 2013-04-24

**Authors:** Silja Raschke, Kristin Eckardt, Kirsten Bjørklund Holven, Jørgen Jensen, Jürgen Eckel

**Affiliations:** 1 Paul-Langerhans-Group of Integrative Physiology, German Diabetes Center, Düsseldorf, Germany; 2 Department of Nutrition, Institute for Basic Medical Sciences, University of Oslo, Oslo, Norway; 3 Department of Physical Performance, Norwegian School of Sport Sciences, Oslo, Norway; Universidad Pablo de Olavide, Centro Andaluz de Biología del Desarrollo-CSIC, Spain

## Abstract

Proteins secreted by skeletal muscle, so called myokines, have been shown to affect muscle physiology and additionally exert systemic effects on other tissues and organs. Although recent profiling studies have identified numerous myokines, the amount of overlap from these studies indicates that the secretome of skeletal muscle is still incompletely characterized. One limitation of the models used is the lack of contraction, a central characteristic of muscle cells. Here we aimed to characterize the secretome of primary human myotubes by cytokine antibody arrays and to identify myokines regulated by contraction, which was induced by electrical pulse stimulation (EPS). In this study, we validated the regulation and release of two selected myokines, namely pigment epithelium derived factor (PEDF) and dipeptidyl peptidase 4 (DPP4), which were recently described as adipokines. This study reveals that both factors, DPP4 and PEDF, are secreted by primary human myotubes. PEDF is a contraction-regulated myokine, although PEDF serum levels from healthy young men decrease after 60 min cycling at VO_2_max of 70%. Most interestingly, we identified 52 novel myokines which have not been described before to be secreted by skeletal muscle cells. For 48 myokines we show that their release is regulated by contractile activity. This profiling study of the human skeletal muscle secretome expands the number of myokines, identifies novel contraction-regulated myokines and underlines the overlap between proteins which are adipokines as well as myokines.

## Introduction

Skeletal muscle is the largest organ in the human body and path-breaking work during the last decade has demonstrated that skeletal muscle is an active endocrine organ releasing a host of so-called myokines [1].

Skeletal muscle exhibits profound adaptability in response to environmental influences. Exercise training enhances muscular endurance [2] and strength [3], expends calories, and leads to a decrease in adipose tissue mass. Additionally, it exerts major beneficial effects on the prevention of chronic diseases such as obesity and type 2 diabetes [4]. These systemic effects of exercise cannot be explained exclusively by the expenditure of calories. It has been suggested that skeletal muscle communicates with other tissues such as liver, adipose tissue, heart, brain, and vasculature by secreting a variety of myokines. Some of these myokines are described to mainly affect muscle physiology in an autocrine fashion, while other myokines additionally exert systemic effects on other tissues and organs. Several myokines are described in the literature to be regulated by contraction, like angiopoietin-related protein 4 [5], fibroblast growth factor 21 [6], interleukin (IL)-6 [7], [8], IL-7 [9], IL-15 [10], leukemia inhibitory factor [11], myonectin [12], myostatin [13] and vascular endothelial growth factor [14], [15]. For some of the reported myokines, only increased mRNA or protein levels in cell or tissue lysates or biopsies are reported, while alterations in serum level after exercise or increased protein levels in supernatants of cultured myotubes after contraction were not reported. In this study, we define a myokine as a protein for which it is clearly proven to be released by skeletal muscle cells.

Boström et al. recently published that PGC1α over-expression in mouse muscle stimulates increased expression of FNDC5, a membrane protein that is cleaved at the N-terminus thereby releasing a soluble protein, called irisin.This protein acts on white adipose tissue-derived adipocytes *in vitro* and *in vivo* to stimulate a broad program of brown-fat-like development [16]. Up to now, IL-6 is the most prominent muscle-derived protein, which was demonstrated to be upregulated in plasma after exercise [8], [17], [18]. IL-6 increases insulin-stimulated glucose disposal in humans and glucose uptake as well as fatty acid oxidation *in vitro* in rat myotubes [19]. Infusion of IL-6 in healthy young men identified IL-6 as potent modulator of fat metabolism in humans, increasing fat oxidation and fatty acid (FA) reesterification [20]. Before this observation, IL-6 was described as a cytokine and adipokine. IL-6 is increased in the plasma of obese patients [21], [22] and overexpressed in human fat cells from insulin-resistant subjects [23]. Additionally, IL-6 has been shown *in vitro* to induce insulin resistance in hepatocytes [24], adipocytes [23] and in skeletal muscle cells after treatment with high doses [25]. The current literature mainly describes a negative crosstalk between excess body fat and skeletal muscle [26], while the data of IL-6 and irisin indicate an additional crosstalk from the muscle to the adipose tissue.

Previously, we have published the secretome of primary human adipocytes and found 44 novel adipokines [27]. Among others, PEDF [28] and DPP4 [29] were described as novel adipokines. Both cytokines are associated with obesity and insulin resistance [28], [29]. Interestingly, we also found these two proteins to be secreted by skeletal muscle cells and termed these proteins adipo-myokines.

To gain a broader view, recent efforts have focused on exploring the complete secretome of skeletal muscle by proteomic studies. Using this approach, Yoon et al. have studied the regulation of protein secretion by rat skeletal muscle cells after insulin stimulation [30], Chan et al. and Henningsen et al. have investigated altered regulation of secretome components during differentiation of murine C2C12 cells [31], [32]. Hittel et al. have explored the secretome of cultured myotubes derived from extremely obese compared with healthy non-obese women [33]. Further, Norheim and colleagues have published the proteomic identification of secreted proteins from human skeletal muscle cells, but regulation of myokines by exercise was only assessed at the mRNA level [34]. A drawback of all these studies is the use of non-contracting cells, although contraction is a major characteristic of skeletal muscle activating intracellular signaling pathways and metabolic adaption.

Recently, we have established and characterized an *in vitro* model of human skeletal muscle cell contraction. Similarly to exercising skeletal muscle *in vivo*, EPS induced contractile activity in human myotubes, accompanied by formation of sarcomeres, activation of AMP-activated protein kinase and increased IL-6 secretion [35]. Thus, EPS application to human skeletal muscle cells represents an excellent tool to study the release of myokines induced by muscle contraction under *in vitro* conditions.

Currently, no biochemical technique exists that can efficiently separate and consistently detect the total protein composition of the cellular secretome, e.g. neither the rodent cell studies [30]–[32], [36], [37], nor the human cell studies [33], [34] identified myokines belonging to the group of interleukins by a proteomic approach. In order to search for proteins undetected by the proteomics approaches, in this study, we have analyzed the secretome of contracting primary human myotubes by cytokine antibody arrays.

## Experimental Procedures

### Materials

Reagents for SDS-PAGE were provided by GE Healthcare (Munich, Germany) and by Sigma (Munich, Germany), rotiphorese was supplied by Carl Roth (Karlsruhe, Germany). The following antibodies were used: anti-DPP4 (Abnova, Heidelberg, Germany), anti-PEDF (Millipore, Darmstadt, Germany), anti-tubulin (Calbiochem/Merck Biosciences, Schmalbach, Germany). Horseradish peroxidase-conjugated goat anti-rabbit and anti-mouse IgG were purchased from Promega (Mannheim, Germany). All other chemicals were of the highest analytical grade commercially available and were purchased from Sigma.

### Culture of Human Skeletal Muscle Cells

Primary human skeletal muscle cells (PromoCell, Heidelberg, Germany) supplied as proliferating myoblasts were cultured as described in our earlier study [38]. Myoblasts of 5 different donors (three males 16, 21, and 47 years old [M16, M21, M47] and two females 33 and 37 years old [W33, W37]) were differentiated to myotubes and used for analysis to take biological variability into account. For an individual experiment, myoblasts were seeded in six-well culture dishes at a density of 10^5^ cells/well and cultured in α-modified Eagle’s (αMEM)/Ham’s F-12 medium containing skeletal muscle cell growth medium supplement pack (PromoCell, Heidelberg, Germany) up to near-confluence. The cells were then differentiated in αMEM containing 2% horse serum (Gibco, Berlin, Germany) until day 5 of differentiation followed by overnight starvation in αMEM without serum after washing with PBS twice.

### EPS

EPS was applied to fully differentiated myotubes in six-well culture dishes using a C-Dish in combination with a C-Pace pulse generator (C-Pace 100, IonOptix, Milton MA). Myotubes were stimulated with a frequency of 1 Hz, 2 ms pulse duration, an intensity of 11.5 V for 4 to 24 h. This protocol rather reflects regular exercise due to unchanged MHCIIa expression [35] and enhanced MHCI expression (K. Eckardt, unpublished data). Cells were washed twice with PBS after overnight starvation and fresh medium was added directly before stimulation. Conditioned medium (CM) was collected after a period of 24 h.

### Immunoblotting

Skeletal muscle cells were treated as indicated and lysed. The immunoblotting procedure was carried out as described before [39]. Signals were visualized on a VersaDOC 4000 MP (Bio-Rad Laboratories, Munich, Germany) and analyzed by Quantity One analysis software (version 4.6.7, Bio-Rad Laboratories).

### RNA Isolation and Quantitative real-time PCR

Total RNA was isolated and reverse transcribed using the RNeasy Mini kit and Omniscript Reverse Transcription kit (Qiagen, Hilden, Germany) according to the manufacturer’s instructions. Gene expression was determined by quantitative real-time PCR using QuantiTect primer assays and SYBR green reagents (Qiagen, Hilden, Germany) with 0.016–20 ng of cDNA on a Step One Plus Cycler (Applied Biosystems, Carlsbad, CA, USA). Expression levels of investigated genes were normalized to actin. Gene expression was analyzed via the ΔCt method.

### Cytokine Antibody Array and ELISA Analysis

Primary human myotubes (4 different donors) were subjected to EPS or left unstimulated, and CM was collected over a period of 24 h to assure that differences of low abundant proteins were in the range of array and ELISA sensitivity. Undiluted CMs were then analyzed by RayBio human cytokine antibody arrays (array #5, #9, #10, Raybiotech, Norcross, GA, USA) according to the manufactures instructions and signals were visualized and evaluated on a LUMI Imager (Boehringer, Mannheim, Germany) work station. Only signals that were above the background and detected in the CM of at least three donors were included in the analysis. For further validation cytokine concentration in CM was determined using DPP4 (R&D Systems) and PEDF ELISA (Biovendor), respectively, both assays used according to the manufacturer’s protocol.

### Human Study

Eight well-trained healthy lean male volunteers (body mass index 23.1±0.6 kg/m^2^, VO_2_max 65.5±1.7 ml/min/kg) participated in the study. A baseline blood sample was taken before start of the exercise. Subjects cycled at 70% VO_2_max for 60 min and then rested for 2 h. Blood was sampled immediately after the exercise session as well as 30 min and 120 min post exercise. Written informed consent was obtained from all the participants. The study was approved by the Regional Committee for Medical and Health Research Ethics, Region Sør-Øst-Norge, Norway (2011/927b).

### Presentation of Data and Statistics

Data are the means ± SEM. Unpaired two-tailed Student t-test or one-way ANOVA (post hoc test Tukey’s multiple comparison test) were used to determine statistical significance. All statistical analyses were performed using Prism5 (GraphPad, LA Jolla, CA) considering a P value of <0.05 as statistically significant. Corresponding significance levels are indicated in the figures.

## Results

### Identification of the Adipokines DPP4 and PEDF as Myokines

**Figure 1:**
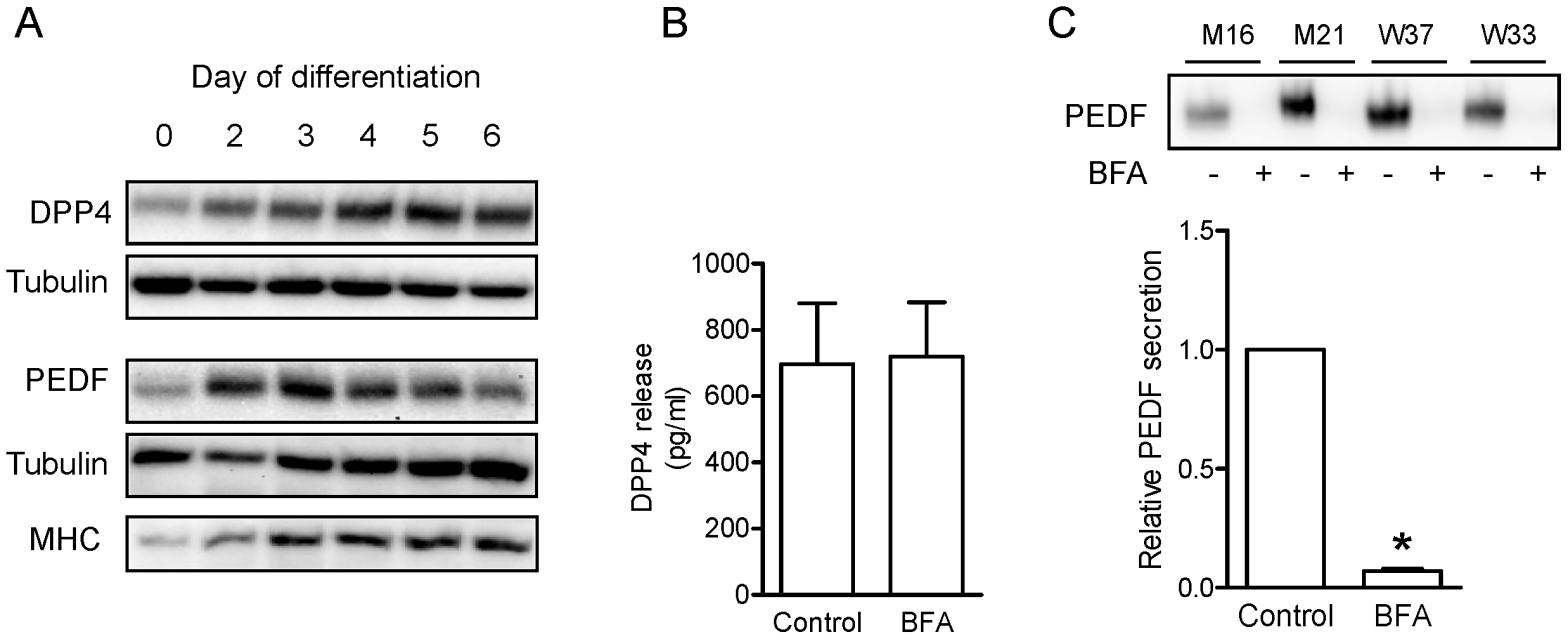
DPP4 and PEDF protein level during differentiation and release by human primary myotubes. A. Primary human myoblasts were differentiated for 0 to 6 days. Protein level of DPP4 and PEDF were analyzed in total cell lysates by SDS-PAGE and Western blot. MHC served as a positive control for differentiation. B+C. To analyze the secretion mechanism, myotubes were incubated with 1 µg/µl BFA for 24 h on day 5 of differentiation. The release of DDP4 was analyzed by ELISA (B, n = 10) and the release of PEDF was analyzed by Western blot (C, n = 6, *p<0.001).

**Figure 2:**
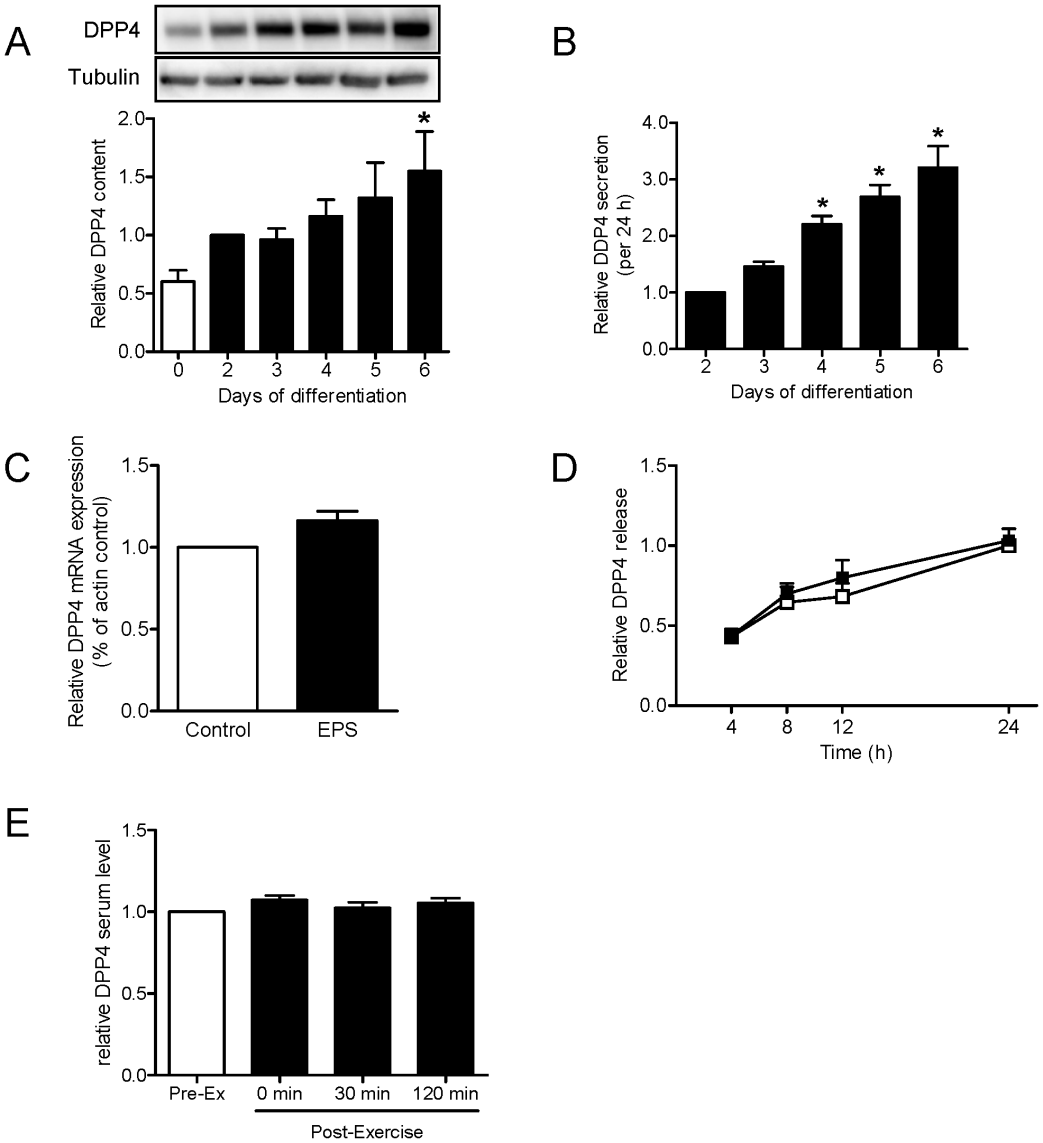
DPP4 protein level and release from myotubes and serum level after acute exercise. A. Primary human myotubes were differentiated and DPP4 protein level during differentiation was analyzed by SDS-PAGE and Western blot. Data were normalized to the protein level of tubulin and are expressed relative to day 2 of differentiation. n = 4, *p<0.05 vs. day 2 of differentiation. B. Release of DPP4 during differentiation of myotubes was analyzed by ELISA. Data were normalized to day 2 of differentiation and are expressed relative to day 2 of differentiation. n = 9−10, *p<0.001 vs. day 2 of differentiation. C. Relative gene expression of DPP4 after 24 h EPS (1 Hz, 2 ms, 11.5 V) compared to non-stimulated cells was measured by real-time PCR as described, n = 4. D. DPP4 released by human myotubes was measured after 4, 8, 12 and 24 hours in non-stimulated cells compared to cells stimulated by EPS using ELISA, n≥11. White symbols, control; black symbols, EPS. E. Serum samples were taken before and after 60 min cycling (70% VO_2_max) at indicated time points. Serum DPP4 was analyzed by ELISA, n = 8. All data are mean values ± SEM.

In this study, we identified the two known adipokines DDP4 and PEDF to be secreted by skeletal muscle cells. The analyses of myocyte lysates confirmed the expression of PEDF and DPP4 in both human myoblasts and myotubes (Fig. 1A). Myocyte differentiation was confirmed by myosin heavy chain (MHC) expression (Fig. 1A).To study intracellular protein transport and to demonstrate weather the proteins are classically secreted, primary human myotubes were incubated with brefeldin A (BFA) for 24 h. BFA interferes with the transport from the endoplasmatic reticulum to the Golgi apparatus [40]. CM was analyzed for DPP4 by ELISA (Fig. 1B) and for PEDF by Western Blot (Fig. 1C), thereby validating the secretion of these factors from primary human myotubes. While DPP4 secretion was unaffected (Fig. 1B), supporting the hypothesis that soluble DPP4 is released by shedding from the cell membrane (reviewed in [41]), BFA inhibited the secretion of PEDF (Fig. 1C). The protein level of DPP4 was analyzed before (day 0 of differentiation) and up to 6 days of differentiation and increased significantly with myogenesis (Fig. 2A). Although secretion did vary between the donors (136.6±26.6–780±52.4 pg/ml on day 2 of differentiation and 592.6±176.2–1850.8±112.5 pg/ml on day 6 of differentiation), relative secretion of DPP4 was significantly upregulated during differentiation (Fig. 2B). Contraction induced by EPS did neither increase DPP4 mRNA expression (Fig. 2C) nor DPP4 secretion (Fig. 2D). DPP4 serum levels from healthy young men were not changed after an acute bout of exercise (Fig. 2E).

**Figure 3:**
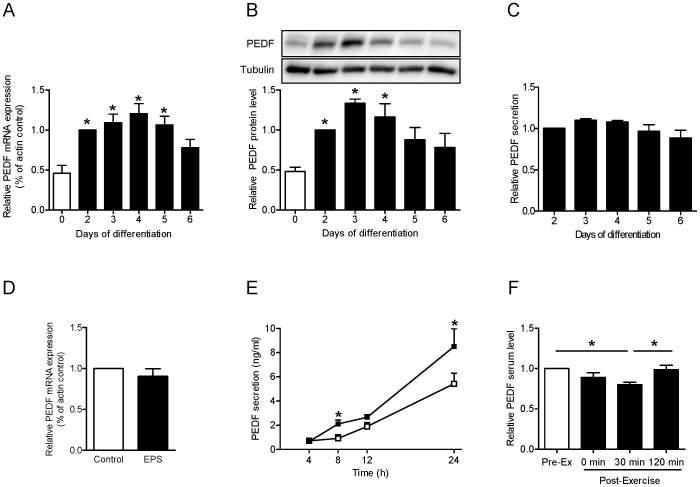
PEDF expression, protein level, and release in myotubes and serum concentration after acute exercise. A. Relative gene expression of PEDF during differentiation of myotubes was measured by real-time PCR as described and is expressed relative to day 2 of differentiation, n = 3−4, *p<0.05 vs. day 0 of differentiation. B. PEDF protein level of primary human myotubes was analyzed during differentiation by SDS-PAGE and Western blot. Data were normalized to the protein level of tubulin and are expressed relative to day 2 of differentiation. n = 5−6, *p<0.05 vs. day 0 of differentiation. C. Secretion of PEDF during differentiation of myotubes was analyzed by ELISA. Data are normalized to day 2 of differentiation, n = 5. D. Relative gene expression of PEDF after 24 h EPS (1 Hz, 2 ms, 11.5V) compared to non-stimulated cells was measured by real-time PCR as described, n = 4. E. PEDF secretion of human myotubes was measured after 4, 8, 12 and 24 hours in non-stimulated cells compared to cells stimulated by ELISA, n = 3−4, *p<0.05 vs. control. White symbols, control; black symbols, EPS. F. Serum samples were taken before and after 60 min cycling (70% VO2max) at indicated time points and PEDF level was analyzed by ELISA, n = 8, *p<0.05. All data are mean values ± SEM.

PEDF mRNA expression and protein level were induced during the first days of myogenesis (Fig. 3A and B) reaching a maximum on day 3 and 4 of differentiation. Secretion of PEDF was not altered during differentiation from day 2 to day 6 (Fig. 3C). Relative mRNA expression was not changed after 24 h EPS (Fig. 3D). Although PEDF secretion from primary human myotubes was significantly enhanced after 8 and 24 h of EPS (Fig. 3E), measurement of PEDF serum level after 60 min cycling revealed a decrease 30 min post exercise (Fig. 3F) that was normalized after 120 min post exercise.

### Identification of Novel Myokines

**Figure 4:**
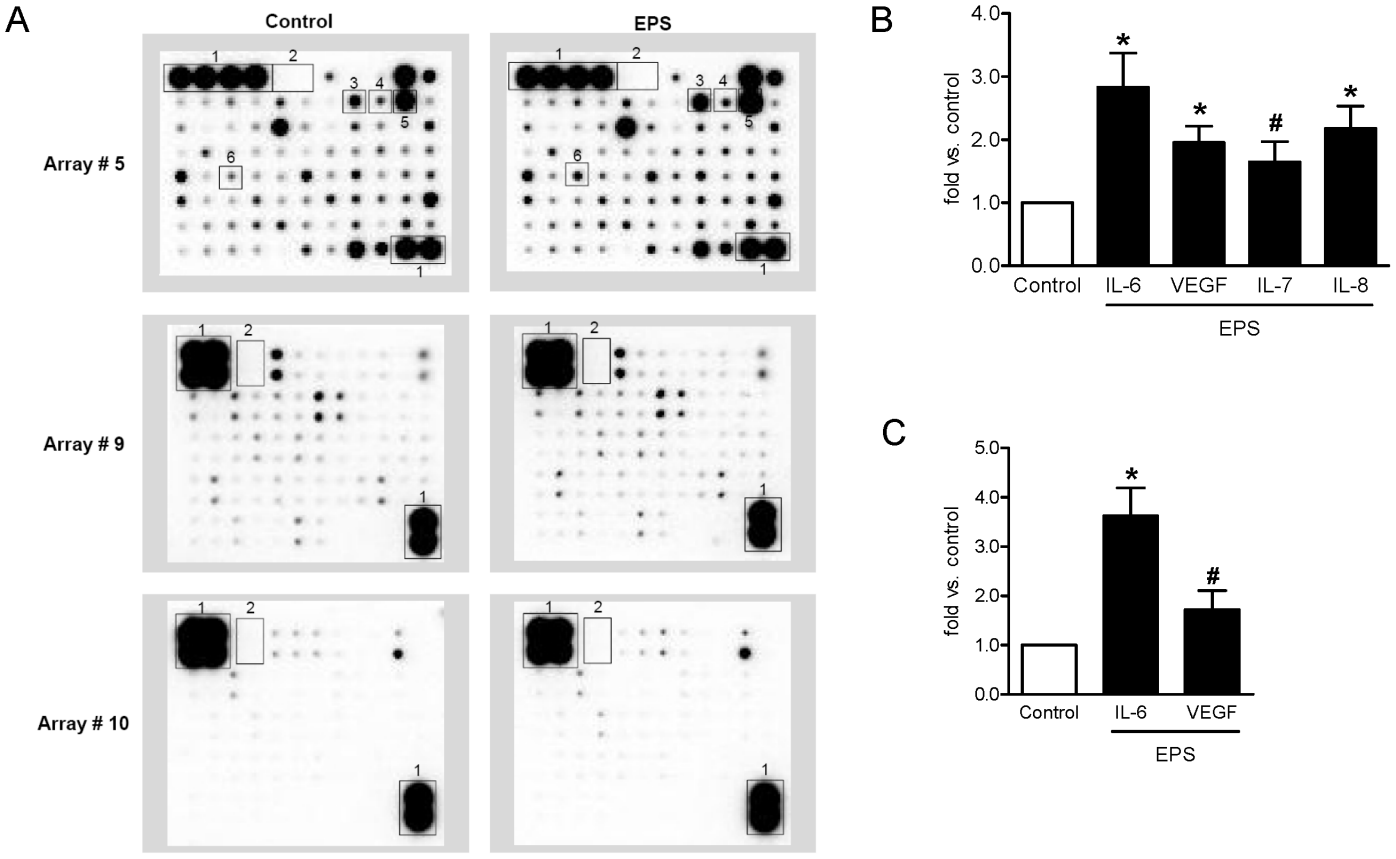
Assessment of contraction-regulated myokines by cytokine antibody arrays. CM of control and EPS-treated cells were collected after 24 h and analyzed as described in Material and Methods. A. Cytokine antibody array membranes #5, #9 and #10 after incubation with CM of control and EPS-treated cells are shown. The encircled areas reflect spots corresponding to the myokines shown in Fig.4B. 1 = positive control; 2 = negative control; 3 = IL-6; 4 = IL-7; 5 = IL-8; 6 = VEGF. B. Known contraction-regulated myokines were analyzed by cytokine antibody arrays and quantified as positive controls. IL, interleukin; VEGF, vascular endothelial growth factor, n = 4, *p<0.01 vs. control, #p<0.05 vs. control. C. IL-6 and VEGF protein concentration were additionally analyzed by ELISA in CM of control and EPS-treated cells. IL-6, n = 6, *p<0.01. VEGF, n = 4, #p<0.05. White bars, control; black bars, EPS.

Using three different cytokine antibody arrays, we analyzed CM obtained from myotubes in order to identify novel proteins secreted by skeletal muscle cells. In total, 179 peptides were analyzed and for 116 of these a positive signal was detected after incubating the membranes with CM obtained from at least three different donors (Fig. 4A, Control). Among the identified peptides 52 factors are novel myokines such as brain-derived neurotrophic factor and somatotropin, for which a release by skeletal muscle cells has not been shown so far (Table 1). In addition, the list emphasizes that beside the known myokines IL-6 and IL-15, further interleukins are secreted such as IL-1α, IL-3, IL-16, IL-22, IL-28a, IL-29 and IL-31 (Table 1).

**Table 1:** Identification of novel myokines by cytokine antibody arrays.

Swiss prot accession	Protein name	Swiss prot accession	Protein name
Q15109	Advanced glycosylation end product-specific receptor	Q14005	Interleukin-16
P23560	Brain-derived neurotrophic factor	Q9GZX6	Interleukin-22
P22362	C-C motif chemokine 1	Q8IZJ0	Interleukin-28A
Q16663	C-C motif chemokine 15	Q8IU54	Interleukin-29
Q92583	C-C motif chemokine 17	P08700	Interleukin-3
P55774	C-C motif chemokine 18	Q6EBC2	Interleukin-31
O00585	C-C motif chemokine 21	P21781	Keratinocyte growth factor
P55773	C-C motif chemokine 23	P21583	Kit ligand
O00175	C-C motif chemokine 24	P01229	Lutropin subunit beta
Q9Y258	C-C motif chemokine 26	P01374	Lymphotoxin-alpha
P13236	C-C motif chemokine 4	P09237	Matrilysin
P01233	Choriogonadotropin subunit beta	Q29983	MHC class I polypeptide-related sequence A
P00746	Complement factor D	Q29980	MHC class I polypeptide-related sequence B
Q07325	C-X-C motif chemokine 9	P22894	Neutrophil collagenase
O43927	C-X-C motif chemokine 13	P20783	Neurotrophin-3
O75078	Disintegrin and metalloproteinase domain-containing protein 11	P34130	Neurotrophin-4
P78536	Disintegrin and metalloproteinase domain-containing protein 17	P13725	Oncostatin M
P08620	Fibroblast growth factor 4	Q99075	Proheparin-binding EGF-like growth factor (HB-EGF)
P10767	Fibroblast growth factor 6	Q9Y336	Sialic acid-binding Ig-like lectin 9
P31371	Fibroblast growth factor 9	P01241	Somatotropin
P49771	Fms-related tyrosine kinase 3 ligand	P40225	Thrombopoietin
P80162	Granulocyte chemotactic protein 2	P01222	Thyrotropin subunit beta
Q13651	Interleukin-10 receptor subunit alpha	O43557	Tumor necrosis factor ligand superfamily member 14
P08833	Insulin-like growth factor-binding protein 1	O14763	Tumor necrosis factor receptor superfamily member 10B
P01579	Interferon gamma	P28908	Tumor necrosis factor receptor superfamily member 8
P01583	Interleukin-1 alpha	P49767	Vascular endothelial growth factor C

CM of myotubes was collected after 24 h and analysed as described using three different cytokine antibody arrays.

In the case of 40 factors, no signal was detected (Table S1) including angiopoietin-related protein 4 (ANGPTL4). This protein was described before as myokine and it was shown to be secreted by human myotubes after treatment with fatty acids [42] via a PPARδ–dependend pathway. While in supernatant of unstimulated cells ANGPTL4 is below the detection limit, only a rather long-term treatment of human myotubes (48 h) with the PPARδ–specific activator GW501516 results in the accumulation of ANGPTL4 in the supernatant [42]. Additionally, Catoire et al. have shown in a one-legged exercise study that target genes of PPAR transcription factors including ANGPTL4 were induced equally in exercising and non-exercising muscle [43]. Although PPARδ is known to be activated by high intensity exercise [44], Catoire et al. have concluded that the increase of plasma free fatty acid levels due to acute exercise activate PPARs and therefore ANGPTL4 [43]. Since in our *in vitro* model no free fatty acids are present in the medium this might explain why we have not detected ANGPTL4 in the supernatant of contracting myotubes.

In addition, 19 cytokines were only detected in the CM obtained from one or two donors and were not included in the analyses.

### Identification of Contraction-regulated Myokines

Human cytokine antibody array analysis was additionally used as a screening method to detect novel contraction-regulated myokines by directly comparing CM obtained from control and EPS-treated myotubes (Fig. 4A). IL-6, IL-7, IL-8, and VEGF were found to be increased in CM of EPS-treated cells compared to control (Fig. 4B). The release of these cytokines is known to be contraction-regulated and served as positive control. To validate the antibody array analysis, the increase of IL-6 and VEGF secretion was additionally determined by ELISA data and showed comparable results (Fig. 4C).

In addition to the four positive controls, 44 factors were significantly regulated by contraction of skeletal muscle cells (Table 2). Fifteen of these were upregulated by EPS more than 1.5 fold such as stromelysin-2 (2.12±0.35), growth-regulated alpha protein (1.88±0.91) and beta-2-microglobulin (1.81±0.54). Furthermore, 18 of the contraction-regulated myokines have not been described before to be secreted by skeletal muscle cells. Another 44 myokines were not regulated by contraction (Table S2). For three factors we observed a significant downregulation in the CM of contracted vs. non-contracted myotubes. Surprisingly, one of these proteins is leukemia inhibitory factor (LIF) which was described before to be upregulated after contraction [45]. In our study we found a reduction of LIF by 14%. These three proteins might be unstable, be degraded by other released factors, or proteins might bind to myotubes leading to internalization again. Future studies should address the detailed description of secretion mechanisms of selected myokines.

**Table 2:** Identification of contraction-regulated myokines using cytokine antibody arrays.

Swissprot accession	Protein Name	Fold vs. Control
P05231	Interleukin-6	2.83 (±0.54)*
P10145	Interleukin-8	2.18(±0.35)*
P09328	Stromelysin-2	2.12 (±0.18)*
P15692	Vascular endothelial growth factor A	1.96 (±0.26)*
P09341	Growth-regulated alpha protein	1.88 (±0.45)^#^
P61769	Beta-2-microglobulin	1.81 (±0.28)*
P05121	Plasminogen activator inhibitor 1	1.73 (±0.38)^#^
Q9UBP4	Dickkopf-related protein 3	1.68 (±0.15)*
P13232	IL-7	1.65 (±0.32)*
**Q29983**	**MHC class I polypeptide-related sequence A**	1.65 (±0.24)*
P05113	Interleukin-5	1.61 (±0.39)^#^
**P01233**	**Choriogonadotropin subunit beta**	1.59 (±0.16)*
**Q99075**	**Heparin-binding EGF-like growth factor**	1.56 (±0.16)*
P13500	C-C motif chemokine 2	1.55 (±0.33)^#^
**O00585**	**C-C motif chemokine 21**	1.53 (±0.20)*
P08253	72 kDa type IV collagenase	1.52 (±0.16)*
P09958	Furin	1.51 (±0.09)*
P01137	Transforming growth factor beta-1	1.50 (±0.18)*
**Q13651**	**Interleukin-10 receptor subunit alpha**	1.39 (±0.06)*
**P00746**	**Complement factor D**	**1.38 (±0.17)^#^**
Q02297	Neuregulin-1	1.37 (±0.16)^#^
P07288	Prostate-specific antigen	1.36 (±0.21)^#^
**Q29980**	**MHC class I polypeptide-related sequence B**	1.36 (±0.13)*
Q92823	Neuronal cell adhesion molecule	1.34 (±0.12)*
**P01241**	**Somatotropin**	1.33 (±0.08)*
**O14763**	**Tumor necrosis factor receptor superfamily member 10B**	1.33 (±0.13)*
P14543	Nidogen-1	1.32 (±0.15)^#^
**P40225**	**Thrombopoietin**	**1.31 (±0.17)^#^**
P50895	Basal cell adhesion molecule	1.30 (±0.06)*
**P22894**	**Neutrophil collagenase**	**1.29 (±0.13)^#^**
P60568	Interleukin-2	1.29 (±0.12)*
P01127	Platelet-derived growth factor subunit B	1.27 (±0.10)*
P02792, P02794	Ferritin (light chain, heavy chain)	1.24 (±0.09)^#^
**P09237**	**Matrilysin**	**1.24 (±0.09)^#^**
**P28908**	**Tumor necrosis factor receptor superfamily member 8**	1.22 (±0.07)*
P17936	Insulin-like growth factor-binding protein 3	1.21 (±0.07)*
**Q16663**	**C-C motif chemokine 15**	**1.21 (±0.10)^#^**
**P01229**	**Lutropin subunit beta**	1.20 (±0.03)*
P14210	Hepatocyte growth factor	1.20 (±0.06)*
P40933	Interleukin-15	1.19 (±0.08)*
**P21781**	**Keratinocyte growth factor**	**1.17 (±0.08)^#^**
P51671	Eotaxin	1.17 (±0.05)*
**P22362**	**C-C motif chemokine 1**	1.16 (±0.07)*
**P10767**	**Fibroblast Growth Factor 6**	1.14 (±0.04)*
**Q6EBC2**	**Interleukin-31**	1.12 (±0.05)*
P15018	Leukemia inhibitory factor	0.86 (±0.06)*
**P80162**	**Granulocyte chemotactic protein 2**	0.77 (±0.08)*
**P34130**	**Neurotrophin-4**	0.76 (±0.06)*

CM of control and EPS-treated myotubes were collected after 24 h and analysed as described. Novel myokines are in bold text. Data are presented as mean fold vs. control (± SEM), n = 3−4, *p<0.01 vs. control, #p<0.05 vs. control (t-test).

## Discussion

Detailed characterization of the human skeletal muscle secretome is essential to understand the beneficial effect of exercise in the context of chronic diseases. Therefore, this study was focused on the identification of myokines released by contracting myotubes.

To consistently detect the total protein composition of the cellular secretome, it is important to combine various technical approaches, since no biochemical technique can efficiently detect all proteins [46]. Although hundreds of secreted proteins were already published by proteomics approaches [9], [14], [30]–[32], 38,45,47–67, our cytokine array approach has identified 52 additional proteins, which we consider as novel myokines secreted by human skeletal myotubes. For several of these factors such as brain derived neurotrophic factor [68], [69], C-C motif chemokine 4 [70], IFNγ [71], [72], neurotrophin-3 [73] and -4 [74], and VEGF-C [75], [76], mRNA expression and/or protein abundance in skeletal muscle tissue or myotubes have been reported, however a clear prove of their release from skeletal muscle cells was not shown. In the case of oncostatin M, indirect evidence of its secretion by myotubes was provided by Hojman et al., however direct assessment of oncostatin M in the supernatant of myotubes is missing [77]. With the cytokine array approach, we could now clearly identify these proteins to be secreted from human skeletal muscle cells.

Secretome analysis showed that the profile of proteins secreted by skeletal muscle changes in response to the treatment with insulin [30], TNFα [37] and during myogenesis [31], [32], [36]. Skeletal muscle is highly adaptable and physical activity exerts a highly complex physiological stimulus triggering multiple biochemical and biophysical aspects of cellular function. Additionally, we found that contraction is another stimulus changing the secretome of myotubes. Overall, our cytokine antibody array analysis revealed that the release of 45 myokines is regulated by contraction. Among these factors, 18 are described as myokines for the first time.

Norheim et al. described the proteomic identification of 236 proteins secreted from non-contracting human skeletal muscle cells and validated the expression of candidates in response to strength training [34]. Using computational analyses the authors distinguished between proteins targeted for export and proteins with specific intracellular retention signals by SignalP and SecretomeP and shortened the list of secreted proteins to 128. To validate expression and activity of secreted proteins on the gene level, Norheim et al. used two additional analyses, a comparison with Human Genome Expression Profile databases together with a published mRNA-based reconstruction of the human skeletal muscle secretome [67]. By this approach, the number of secreted proteins was diminished to 18 classically secreted proteins. This strategy ensures that proteins are secreted by skeletal muscle cells and it narrows down the number of clearly, high abundant targets. However, this strategy might imply the loss of physiological important targets. The mRNA-based reconstruction of the muscle secretome included proteins putatively secreted based on the presence of a predicted signal peptide and the absence of predicted transmembrane regions [67]. This excludes all proteins which are secreted by shedding, the proteolysis of ectodomains of membrane proteins, like the novel myokine irisin [16] and most likely DPP4 [78].

### Validation of Novel Myokines

One myokine we have analyzed in this study is PEDF, which is a 50 kDa secreted glycoprotein that belongs to the non-inhibitory serpin group [79]. PEDF was initially purified from conditioned media of human pigment epithelial cells of the retina and identified as a differentiating factor for retinoblastoma cells [80], [81]. PEDF serum levels are increased in subjects with metabolic syndrome [82] and in type 2 diabetic patients [83]. Insulin resistance is associated with elevated PEDF serum levels in morbidly obese patients [84], while serum PEDF was declined significantly after weight loss in individual patients [84], [85]. All these data indicate that PEDF secreted from adipose tissue is associated with the metabolic syndrome. Despite these data, we found that an acute bout of exercise decreased PEDF serum levels, which was just recently described by Oberbach et al. as well [86]. Nevertheless, PEDF was detected as myokine by us and other secretome studies [32], [34], [67]. Most interestingly, we found that PEDF secretion of primary human myotubes is increased after contraction induced by EPS, although mRNA level was not changed. However, the acute bout of *in vivo* exercise might be better mimicked by short-term, high frequency EPS, as recently described by Nikolić et al. [87]. Additional experiments using this approach will be required to explore the contraction-regulated regulated release of PEDF from human myotubes. Further, Norheim et al. showed that post-exercise to strength training transcription of muscle PEDF was increased in humans [34], indicating that the upregulation of PEDF in response to exercise is rather a long-term effect. A query using BioGPS, a gene annotation portal, indicates that PEDF is mainly expressed in adipose tissue, liver and retina [88]. PEDF is one of the most abundant proteins of primary adipocytes, and myotubes secrete PEDF at significantly lower concentrations compared to preadipocytes and adipocytes [28]. Thus, it might be speculated that serum PEDF levels mainly originate from adipose tissue, and PEDF secreted from skeletal myotubes upon contraction acts rather in an autocrine/paracrine manner within the muscle.

The second validated myokine is DPP4, a cell surface type II membrane glycoprotein. A BioGPS query revealed that DDP4 is ubiquitously expressed. DPP4 cleaves N-terminal dipeptides of polypeptides like glucagon-like peptide 1 (GLP1) and glucose dependent insulinotropic polypeptide (GIP), two post-prandial activated incretins [89]. Insulin secretion is enhanced and glucose tolerance is improved in mice lacking DPP4 [90]. Therefore, DPP4 has gained considerable interest as a therapeutic target, and a variety of DPP4-inhibitors which enhance glucose-dependent insulin secretion from pancreatic ß-cells are now in clinical use as anti-diabetic drugs [91].

Substantial DPP4 activity is also found in plasma because of a soluble form of DPP4 lacking the cytoplasmic tail and the transmembrane region of this protein. BioGPS query revealed that DPP4 is predominantly expressed in the kidney, smooth muscle cells, cardiac myocytes, prostate and small intestine. However, the major source of circulating DPP4 and its regulation remain unknown. By using adipose tissue explants from lean and obese subjects, Lamers et al. observed an increase in DPP4 serum levels that strongly correlated with adipocyte volume and parameters of the metabolic syndrome, while DPP4 serum levels decreased to the lean level after weight reduction [29], [78]. Since obesity is correlated with a low grade systemic whole body inflammation, we tested inflammatory cytokines to influence DPP4 secretion from myotubes. DPP4 secretion was not altered by inflammatory cytokines like TNFα, MCP-1, IFNγ and IL-6 as well as insulin (S. Raschke, unpublished observation). Additionally, contractile activity of myotubes did not change DPP4 secretion over 24 h. We could confirm that DPP4 is not classically secreted, since BFA did not change soluble DPP4 concentration in the CM of myotubes. DPP4 function of the soluble form remains poorly understood, and it is unknown if the process of DPP4 release from cell membranes is regulated or not, and this should be addressed by future studies.

### Contraction-regulated Myokines

Most interestingly, we compared the CM of control and contracting myotubes by cytokine antibody array analysis. Among others, C-C motif chemokine 2 (MCP-1) and somatotropin were identified as contraction-regulated myokines.

MCP-1 resembles IL-6, which was long-time only recognized as inflammatory cytokine and then found to be an exercise factor with a positive impact on muscle physiology [92]. MCP-1 is a chemokine and member of the small inducible cytokine family and plays a crucial role in the recruitment of monocytes and T lymphocytes into tissues [93], [94]. It is produced in primary adipocytes among other cell types and associated with obesity [95]. MCP-1-induced macrophage infiltration in adipose tissue leads to a chronic state of low-grade inflammation [96], which is linked to insulin resistance. Additionally, *in vitro* data demonstrate this factor has the ability to induce insulin resistance in adipocytes and skeletal muscle cells [97]. Here, we describe that MCP-1 is a contraction-regulated myokine. Increased MCP-1 mRNA expression in skeletal muscle was reported by Hubal et al. after a repeated eccentric exercise bout [98]. Garcia et al. showed an increase of circulating MCP-1 levels in healthy sedentary women after a single session of exercise [99]. In addition, a study using cultured human skeletal muscle cells revealed that mechanical strain enhanced the release of MCP-1 by these cells [49]. Future studies have to further characterize the effects of exercise on systemic and muscle MCP-1 and its potential autocrine/paracrine effects on skeletal muscle that may be related to adaptation processes.

Somatotropin is a pleiotropic peptide hormone with an important role in the regulation of metabolism via stimulation of lipid mobilization and oxidation [100]. It is well known that physical exercise increases somatotropin level in healthy subjects and there is also evidence that the acute somatotropin response to exercise is important in regulating fatty acid availability in the post exercise setting [101]. Several studies investigated the anabolic effects of somatotropin on skeletal muscle cells showing an increase of IGF-1 expression [102], protein synthesis [103], and the size of differentiated myotubes [104]. However, to the best of our knowledge we are the first to show that somatotropin is secreted by myotubes and regulated by contraction.

### Adipo-Myokines

This study now revealed that there is a considerable overlap between the adipokines and myokines. A considerable number of secreted proteins from skeletal muscle are also secreted by adipocytes. We defined these proteins as adipo-myokines. Among others, DPP4 [29], PEDF [28] as well as MCP-1 [97], decorin [105] and PAI-1 [106] are described to be associated with conditions of obesity, insulin resistance and diabetes. Therefore, up to now, these cytokines were described as adipokines in the literature. Myokines itself might not only have autocrine and/or paracrine effects in the tissue of origin, several of them might also be distributed by the circulation and affect other tissues in an endocrine manner. Thereby, these adipo-myokines might participate in a bi-directional crosstalk between skeletal muscle and adipose tissue. The physiological role of adipo-myokines may vary depending whether the cytokines act in an autocrine and endocrine manner as well as in a chronic or acute manner. In healthy, normal weight subjects skeletal muscle is the largest tissue in the human body, accounting for 40−50% of total human body mass, while body fat accounts for 20−35%. In obese subjects the percentage of total body fat increases to 40−60% resulting in an increased secretion of pro-inflammatory adipokines, while the percentage of skeletal muscle is decreased and so might the myokine serum level. The aim of future studies will be to identify adipo-myokines that are detectable in the circulation, to determine their origin and to analyze their role in the crosstalk between skeletal muscle and adipose tissue in the obese state and after exercise.

The present study contributes to the understanding of the endocrine function of human myotubes. Secretory proteins are part of a complex physiological network, and they exert different effects under various environmental conditions. Therefore, our current knowledge of contraction-regulated secretory proteins from skeletal muscle and their roles in distinct signaling pathways according to the organ has to be determined in future studies. For novel myokines presented in this study, additional basic research and clinical studies will provide a further understanding in exercise metabolism.

## Supporting Information

Table S1 **Cytokines that were not detected in CM of hSkMC using cytokine antibody arrays.** CM of control and EPS-treated myotubes were collected after 24 h and analysed as described. This list contains all cytokines that did not produce a signal above the background.
(DOCX)

Table S2 **List of Myokines that were not regulated by contraction.** CM of control and EPS-treated myotubes were collected after 24 h and analysed as described.
(DOCX)
