# Supplementary material for: Identification and Validation of Novel Contraction-Regulated Myokines Released from Primary Human Skeletal Muscle Cells
Source: PLoS One. 2013 Apr 24;8(4):e62008. doi: 10.1371/journal.pone.0062008 (PMC3634789; doi:10.1371/journal.pone.0062008)
Supplement: Table S1 — Cytokines that were not detected in CM of hSkMC using cytokine antibody arrays. CM of control and EPS-treated myotubes were collected after 24 h and analysed as described. This list contains all cytokines that did not produce a signal above the background. (DOCX) [file pone.0062008.s001.docx]

| **Swissprot accession** | **Protein Name** |
| --- | --- |
| Q9BY76 | Angiopoietin-related protein 4 |
| P00747 | Angiostatin |
| Q16790 | Carbonic anhydrase 9 |
| P13688 | Carcinoembryonic antigen-related cell adhesion molecule 1 |
| Q16627 | C-C motif chemokine 14a |
| P29965 | CD40 Ligand |
| O94907 | Dickkopf-related protein 1 |
| Q9UBT3 | Dickkopf-related protein 4 |
| Q92838 | Ectodysplasin-A |
| P16422 | Epithelial cell adhesion molecule |
| P47929 | Galectin-7 |
| P09919 | Granulocyte colony-stimulating factor |
| Q96D42 | Hepatitis A virus cellular receptor 1 |
| P01308 | Insulin |
| P35225 | Interleukin-13 |
| Q9UHF5 | Interleukin-17B |
| Q9P0M4 | Interleukin-17C |
| Q96PD4 | Interleukin-17F |
| Q96F46 | Interleukin-17 receptor A |
| P31994 | Low affinity immunoglobulin gamma Fc region receptor II-b |
| Q9Y5Y7 | Lymphatic vessel endothelial hyaluronic acid receptor 1 |
| Q14108 | Lysosome membrane protein 2 |
| P13591 | Neural cell adhesion molecule 1 |
| P41271 | Neuroblastoma suppressor of tumorigenicity 1 |
| Q07326 | Phosphatidylinositol-glycan biosynthesis class F protein |
| P02776 | Platelet Factor 4 |
| P01258 | Procalcitonin |
| P58294 | Prokineticin-1 |
| P04271 | Protein S100-B |
| P04626 | Receptor tyrosine-protein kinase erbB-2 |
| Q9BQR3 | Serine protease 27 |
| Q15465 | Sonic hedgehog protein N-product |
| P13385 | Teratocarcinoma-derived growth factor 1 |
| Q969D9 | Thymic stromal lymphopoietin |
| P01266 | Thyroglobulin |
| Q9NP99 | Triggering receptor expressed on myeloid cells 1 |
| Q02223 | Tumor necrosis factor receptor superfamily member 17 |
| P25942 | Tumor necrosis factor receptor superfamily member 5 |
| P62979 | Ubiquitin-40S ribosomal protein S27a |
| P19320 | Vascular cell adhesion protein 1 |
