# Supplementary material for: Identification and Validation of Novel Contraction-Regulated Myokines Released from Primary Human Skeletal Muscle Cells
Source: PLoS One. 2013 Apr 24;8(4):e62008. doi: 10.1371/journal.pone.0062008 (PMC3634789; doi:10.1371/journal.pone.0062008)
Supplement: Table S2 — List of Myokines that were not regulated by contraction. CM of control and EPS-treated myotubes were collected after 24 h and analysed as described. (DOCX) [file pone.0062008.s002.docx]

| **Swissprot accession** | **Protein Name** | **Swissprot accession** | **Protein Name** |
| --- | --- | --- | --- |
| Q15109 | Advanced glycosylation end product-specific receptor | P22301 | Interleukin-10 |
| P02771 | Alpha Fetoprotein | P29459, P29460 | Interleukin-12 p70 (subunit p35, subunit p40) |
| P03950 | Angiogenin | Q14005 | Interleukin-16 |
| P23560 | Brain-derived neurotrophic factor | P01583 | Interleukin-1 alpha |
| Q99616 | C-C motif chemokine 13 | P01584 | Interleukin-1 beta |
| Q92583 | C-C motif chemokine 17 | Q9GZX6 | Interleukin-22 |
| P55774 | C-C motif chemokine 18 | Q8IZJ0 | Interleukin-28A |
| P55773 | C-C motif chemokine 23 | Q8IU54 | Interleukin-29 |
| O00175 | C-C motif chemokine 24 | P08700 | Interleukin-3 |
| Q9Y258 | C-C motif chemokine 26 | P05112 | Interleukin-4 |
| P13236 | C-C motif chemokine 4 | P21583 | Kit ligand |
| P13501 | C-C motif chemokine 5 | P41159 | Leptin |
| P80098 | C-C motif chemokine 7 | P01374 | Lymphotoxin-alpha |
| P80075 | C-C motif chemokine 8 | P09603 | Macrophage colony-stimulating factor 1 |
| P78556 | C-C motif chemokine 20 | P14174 | Macrophage migration inhibitory factor |
| Q07325 | C-X-C motif chemokine 9 | P01033 | Metalloproteinase inhibitor 1 |
| P02778 | C-X-C motif chemokine 10 | P16035 | Metalloproteinase inhibitor 2 |
| O43927 | C-X-C motif chemokine 13 | Q99733 | Nucleosome assembly protein 1-like 4 |
| P42830 | C-X-C motif chemokine 5 | P20783 | Neurotrophin-3 |
| O75078 | Disintegrin and metalloproteinase domain-containing protein 11 | P13725 | Oncostatin M |
| P78536 | Disintegrin and metalloproteinase domain-containing protein 17 | P10451 | Osteopontin |
| P08620 | Fibroblast growth factor 4 | P01133 | Pro-epidermal growth factor |
| P31371 | Fibroblast growth factor 9 | Q9HD89 | Resistin |
| P49771 | Fms-related tyrosine kinase 3 ligand | Q9Y336 | Sialic acid-binding Ig-like lectin 9 |
| P19883 | Follistatin | P48061 | Stromal cell-derived factor 1 |
| P78423 | Fractalkine | P61812 | Transforming growth factor beta-2 |
| Q99988 | Growth/differentiation factor 15 | P10600 | Transforming growth factor beta-3 |
| P39905 | Glial cell line-derived neurotrophic factor | Q15582 | Transforming growth factor-beta-induced protein ig-h3 |
| P04141 | Granulocyte-macrophage colony-stimulating factor | P01375 | Tumor necrosis factor |
| P08833 | Insulin-like growth factor-binding protein 1 | O43557 | Tumor necrosis factor ligand superfamily member 14 |
| P18065 | Insulin-like growth factor-binding protein 2 | O00300 | Tumor necrosis factor receptor superfamily member 11B |
| P22692 | Insulin-like growth factor-binding protein 4 | Q9HAV5 | Tumor necrosis factor receptor superfamily member 27 |
| P05019 | Insulin-like growth factor 1 | P01222 | Thyrotropin subunit beta |
| P01579 | Interferon gamma | P49767 | Vascular endothelial growth factor C |
